# Exercise interventions in child and adolescent mental health care: An overview of the evidence and recommendations for implementation

**DOI:** 10.1002/jcv2.12031

**Published:** 2021-09-18

**Authors:** Rebekah Carney, Joseph Firth

**Affiliations:** ^1^ Youth Mental Health Research Unit Greater Manchester Mental Health NHS Foundation Trust Manchester UK; ^2^ Division of Psychology and Mental Health University of Manchester Manchester UK; ^3^ NICM Health Research Institute Western Sydney University Westmead New South Wales Australia

**Keywords:** adolescence, comorbidity, early intervention, health, intervention

## Abstract

**Background:**

The use of physical activity interventions in mental health care for adults has a large academic evidence base and numerous examples of real‐world implementation. However, the use of physical activity within mental health care for children and young people (CYP) has received less attention to date.

**Methods:**

A narrative review was conducted to summarize the relevant literature in the area. Online databases were searched using terms synonymous with CYP, exercise, physical health, and mental health. Findings from existing systematic reviews, meta‐analyses, meta‐syntheses, and consensus statements were reviewed, and used alongside the authors' experience to inform clinical recommendations.

**Results:**

We first discuss the importance of applying physical health interventions in early stages of mental illness for CYP to prevent physical comorbidities and premature mortality in the long term. We then provide a brief summary of the current evidence of the benefits of exercise interventions in CYP with mental illness. We then present our top five recommendations on the implementation of exercise interventions within CYP mental health care.

**Conclusion:**

The key conclusions from this suggest there is an increasingly strong evidence base for the benefits of using physical activity interventions to improve, prevent, and manage physical and mental health outcomes in CYP with mental illness. However, more work needs to be done to improve the evidence base, refine its implementation into standard mental health care, and develop strategies for large‐scale dissemination of such interventions across various care and cultural contexts.

## Graphical abstract

There is an increasingly strong evidence base for the benefits of using physical activity interventions to improve physical and mental health outcomes in CYP with mental illness. However, more work needs to be done to improve this evidence, refine its implementation into standard mental healthcare, and develop strategies for large‐scale dissemination of such interventions across various care and cultural contexts.

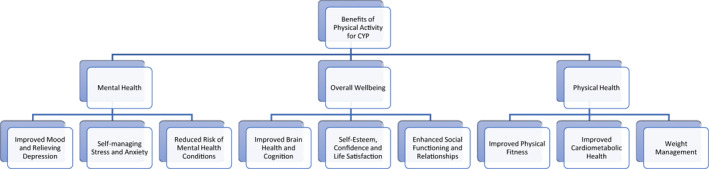

**Key points**

1. The use of physical activity interventions in mental health care for adults has a large academic evidence base; however, the use of physical activity within mental health care services for children and young people has received less attention to date
2. There is an increasingly strong evidence base for the benefits of using physical activity interventions to improve physical and mental health outcomes in CYP with mental health conditions, with the strongest evidence suggesting exercise can protect against depression
3. Exercise is a safe, and relatively cost‐effective intervention and more work needs to be done to identify effective ways to embed such interventions in mental health care

## INTRODUCTION

The poor physical health of people with mental illness has long been established. A 15–20‐year mortality gap arises from factors such as the likelihood of developing noncommunicable diseases, unhealthy lifestyle behaviors, reduced access to and provision of physical health care, and side‐effects of medication (Correll et al., 2017; De Hert et al., 2009; Liu et al., 2017; World Health Organization [WHO], 2018). This has been labeled an international human rights scandal, since a large proportion of this risk is preventable (Maj, 2009; Thornicroft, 2011). In recognition of these disparities, international health bodies have produced guidance to address poor physical health; including the World Health Organization (WHO, 2018), World Psychiatric Association (Liu et al., 2017), and a Lancet Psychiatry Commission (Firth et al., 2019).

Each of these guidelines include recommendations for promoting health behaviors (such as physical activity) for people with severe mental illness (SMI), as a strategy toward reducing the risk of cardiometabolic diseases and associated mortality. In particular, physical activity and exercise interventions may be included as a core component of physical health care from illness onset in young people with SMI, to protect against the development of future noncommunicable diseases. Along with physical health benefits, studies have shown that increasing physical activity can reduce the risk of developing various mental health conditions (Firth, Schuch, et al., 2020; Firth, Solmi, et al., 2020), while also acting as an effective adjunctive intervention for reducing symptoms in those living with mental illness (Ashdown‐Franks et al., 2020). Additionally, increasing physical activity can also seek to manage and alleviate any existing physical health comorbidities such as obesity and chronic pain which may be present and exacerbated by poor mental health. However, compared with the extensive literature on adult populations, less attention has been focused on children and young people (CYP).

## AIMS AND METHODS

The aim of this piece is to provide a succinct overview of the current evidence regarding the importance of addressing physical health in CYP with mental illness, and to examine the existing literature around the role of physical activity and exercise for improving outcomes in this population. This will be used to produce some top recommendations based on existing literature, regarding the real‐world implementation of exercise interventions in CYP mental health settings.

To provide this overview, a narrative review methodology was applied to summarize the key literature and findings on this topic to date. As the aim of this piece was to provide a concise overview of the evidence base and apply this to current clinical practice, a nonsystematic review was undertaken. To identify key relevant studies, relevant online databases such as Ovid, Medline and PubMed, and Google Scholar were searched using terms synonymous with children and young people, exercise, physical health, and mental health. The evidence used to inform this review consisted of peer‐reviewed published articles on the topic of exercise or physical activity interventions for young people with mental health disorders. In particular, the findings from existing systematic reviews, meta‐analyses and meta‐syntheses, and consensus statements were reviewed and used alongside the authors' experience to inform clinical recommendations put forward here. Following this, a narrative synthesis of this information was produced to summarize current understanding on the effects and implementation of physical activity interventions with relevance to child and adolescent mental health.

## RESULTS AND DISCUSSION

### The importance of physical health for CYP with mental health conditions

Half of all mental health conditions appear by the age of 14, and 75% by the age of 25 (England, 2015). As many as 25% of young people will have a diagnosed mental health condition worldwide (Patel et al., 2007; World Health Organization, 2020). In the UK in 2020, 27.2% of young women and 13.3% of young men aged 17–22 had a diagnosed mental health disorder; a rate which continues to rise (NHS Digital, 2020). These prevalence rates also do not consider the many more CYP who experience mental distress, low mood, and anxiety and who do not access mental health services. The Five Year Forward View for Mental Health 2016 included a pledge to improve treatment for CYP by 2021, emphasizing that both the mental and physical health of CYP should be considered a priority.

Young people with mental health conditions exhibit signs of poor physical health from the early stages of diagnosis/treatment of mental illness. There is evidence to suggest that CYP within the early stages of mental health disorders, and even those who are at‐risk (or in “prodromal” stages), have significantly poorer physical health compared with their peers (Carney et al., 2016, 2021; Galling & Correll, 2015). Cardiometabolic risk factors such as increased weight and prevalence of obesity, metabolic diseases, and other co‐morbid physical health conditions (such as dyslipidemia and hypertension) are all observed in CYP with a range of mental health disorders. These physical health problems often go undetected and therefore untreated (Eapen & John, 2011). Furthermore, CYP with mental health disorders are at increased risk for engaging in behaviors which are detrimental to their physical health, such as tobacco smoking, alcohol consumption, unhealthy diet, and physical inactivity, compared with their peers (Carney et al., 2016, 2021; Eapen & John, 2011). Relatedly, CYP with mental health disorders also experience significantly more barriers to living a healthy, active lifestyle, than CYP without mental health disorders. This often includes factors relating to mental health symptoms such as low motivation, lack of energy, social withdrawal, and increased anxiety, as well as specific environmental factors such lack of access to safe outdoor space and facilities (Carney, Cotter, et al., 2017; Carney, Yung et al., 2017; Firth et al., 2016; Rosenbaum et al., 2016).

In some CYP, the need to intervene is even more crucial. Individuals who receive inpatient care have additional vulnerabilities given the “obesogenic” nature of the environment. This refers to factors which increase the likelihood of weight gain, such as increased restrictions such as movement, lack of access to outdoor space and community facilities, less control over dietary intake, fewer opportunities to be active, and increased likelihood of polypharmacy (Carney et al., 2021; Galling & Correll, 2015; Gorczynski et al., 2013; Johnson et al., 2018). Similarly, many CYP with mental health disorders are prescribed psychotropic medications which can further impact on physical health and associated behaviors, because of side effects these may have on increased appetite, lethargy and a reduction in energy, and weight gain (Carney et al., 2018; Eapen & John, 2011; Galling & Correll, 2015; Pillay et al., 2018).

This raises important clinical implications and suggests a need to intervene early to prevent the onset of more severe physical health conditions in the future.

### Benefits of exercise for CYP

**FIGURE 1:**
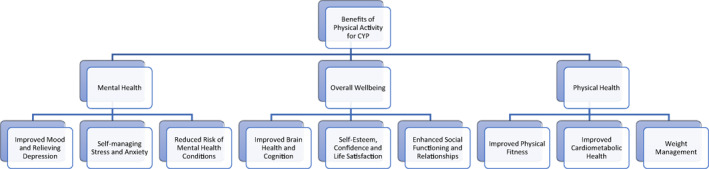
Benefits of exercise for children and young people

Engaging in physical activity has many benefits for CYP (Figure 1). Structured exercise has been shown to have significant mental health benefits, with substantial bodies of evidence showing that exercise can effectively reduce symptoms of depression and improve self‐esteem in CYP (see Dale et al., 2019; Pascoe et al., 2020 for a comprehensive overview). Young people who have higher levels of physical activity also have higher levels of well‐being and quality of life, increased life satisfaction, and are less likely to be diagnosed with a mental health condition. As well as improving symptoms of poor mental health during adolescence, there is also evidence that exercise exerts a protective effect and may reduce the incidence of mental illnesses such as depression and psychosis in the future (Beauchamp et al., 2018; Firth, Schuch, et al., 2020; Firth, Solmi, et al., 2020; Pascoe & Parker, 2019).

As noted by the World Health Organization (WHO), the benefits of exercise for CYP are far reaching. Some of the notable benefits include improved physical fitness, improved cardiometabolic health (blood pressure, glucose, and insulin resistance), better bone health, ability to maintain a healthy weight, improved sleep, and higher cognitive functioning, such as academic performance, executive functioning, and brain health (Bull et al., 2020). Partaking in regular exercise during childhood and adolescence also has significant social benefits, as well as improving self‐perception and identify, quality of life, confidence in ability, and self‐efficacy (Eime et al., 2013). Engaging in community‐based sports has also been recommended to enhance psychological health and social outcomes (Eime et al., 2013). Similarly, the WHO recognizes that a lack of physical activity during childhood can have a detrimental effect on long‐term physical and mental health development, and advocate for a “life course” approach to health (Jacob et al., 2017).

### Interventions to improve physical activity for CYP with mental health disorders

Previous meta‐analyses have demonstrated the utility of exercise interventions in a range of clinical and preclinical populations. This has resulted in extensive evidence of the benefits of exercise interventions for a broad spectrum of mental disorders (Ashdown‐Franks et al., 2020; Firth, Schuch, et al., 2020; Firth, Solmi, et al., 2020).

Despite this, comparably few high‐quality evidence syntheses of RCTs have been conducted with young people. To date, most of the evidence comes from studies targeting depression in CYP (Bailey et al., 2018; Carter et al., 2016). However, two recent scoping reviews synthesized existing systematic reviews of exercise interventions for CYP across a range of mental health conditions (see Biddle et al., 2019; Pascoe et al., 2020). Interventions consisted of predominantly moderate‐vigorous aerobic exercise, targeting depressive symptoms but inclusive of a range of mental health conditions. Exercise interventions showed some efficacy for improving depressive symptoms, self‐esteem, social functioning, cognition, mood states, quality of life, eating disorder symptoms, and achieving remission of anxiety (Biddle et al., 2019; Pascoe et al., 2020). As noted, the strongest evidence was for depression, and comparably less research focus on conditions such as anxiety, stress, and psychosis. Most research conducted has focused on the use of high‐moderate intensity exercise (such as aerobics and sports). However, light‐moderate intensity exercise interventions (such as yoga, walking, and lower intensity exercise) have displayed preliminary evidence to suggest these activities may also have a positive effect on mood and well‐being for CYP (Carter et al., 2015; Hagen & Nayar, 2014).

Interest in this area is growing and future work should seek to identify how interventions to promote physical activity can best be embedded and implemented in mental health services across different settings (such as community based services, secure forensic services, and primary care). Further attention should be given to population groups which have been largely under‐researched including CYP on mental health inpatient units, including forensic services (Carney et al., 2020), young people with emerging psychosis and bipolar disorder (Carney et al., 2016; Firth et al., 2016; Lin et al., 2015), and young people with anxiety disorders and preclinical anxiety (De Silva et al., 2018; Stubbs et al., 2017).

### Recommendations for implementation

Exercise is a relatively simple, safe, and cost‐effective intervention, which can be used in conjunction with comparable evidence‐based treatments such as psychological therapies, (which can be costly and labor intensive) or psychotropic medication (which carries significant metabolic side‐effects). Additionally, CYP have expressed a desire for such treatment options to be used in “youth‐friendly” primary care (Tylee et al., 2007). While it is important for services to continue providing standard evidence‐based treatments, exercise interventions should be offered as a potential adjunct to standard treatments. We recommend embedding such interventions in CYP mental health services and considering the following (Table 1):

1. A comprehensive set of standards should be developed to enable service providers to embed physical activity interventions into routine mental health services. In the UK, National Institute for Health and Care Excellence recommend the use of exercise for young people with depression. However, no formal guidance has yet been established for young people with other mental health conditions and we are unsure of the consistency of application of this approach for CYP with depression (Lawton & Moghraby, 2016). Policy makers, academics, clinicians, and CYP should work together to create a framework for increasing activity levels across CYP mental health services
2. A prevention‐focused approach should be adopted. Rather than waiting to administer health behavior change in response to physical comorbidities arising, further efforts should be invested toward providing such interventions at the earliest possible stage. This involves targeting wide‐reaching community‐based child and adolescent mental health services, using public health initiatives, as well as looking at those providing specialist inpatient care to people already exhibiting signs of ill‐health or already prescribed psychotropic medications with substantial metabolic side‐effects (Carney et al., 2021; Eapen & John, 2011; Galling & Correll, 2015)
3. Accredited exercise professionals should be used to deliver interventions, where possible. Previous research has shown interventions delivered by exercise professionals are more effective, delivered more efficiently, and are more acceptable to service users (Fibbins et al., 2019; Kleemann et al., 2020; Stubbs et al., 2014). Exercise professionals also have specialist training which enables any treatment to be guided by health behavior change principles (Stubbs et al., 2014)
4. Interventions should be flexible, individualized, and sensitive to the needs and motivations of the young person, including being culturally appropriate. Individualized interventions which allow the person to select from a range of activities (such as aerobics, sports) are more beneficial, have higher rates of adherence, and better long‐term outcomes, than a “one size fits all” approach (Ashdown‐Franks et al., 2020; Carter et al., 2017; Firth et al., 2019). This results in increased self‐efficacy via greater mastery of the activity, increased participant‐driven performance, higher levels of enjoyment, and a greater sense of autonomy over the behavior (Pascoe et al., 2020)
5. The setting of the intervention should be appropriate. For example, interventions conducted in outdoor spaces may be more effective for young people with anxiety, than those that take place indoors (Yang et al., 2015). Similarly, group‐based activities confer wide reaching social benefits compared to individual activities (Eime et al., 2013). Consideration should be given to more specialized mental health services such as inpatient secure mental health units, which are often highly restrictive in relation to free movement, meaning activities need to be predominately ward‐based (Carney et al., 2020). On the other hand, the use of community‐based initiatives should be considered via social prescribing, increased access to green and blue space, and use of holistic interventions from outside of mental health services (Chatterjee et al., 2018). To promote long‐term adherence, including others as part of a support network should be considered such as family members and care providers

**TABLE 1:** Top five recommendations for implementation

	Recommendation	Example of real‐world application
1	Development of guidelines to embed physical activity interventions in mental health services.	A mental health service (e.g., Tier 4 CAMHS), has decided to work with service users, family members/carers, clinicians, policy makers, academics, and other experts to develop standards which can be used to guide the promotion of physical activity within the care of all young people treated within their service.
		This may include training of staff in physical health monitoring and promotion, use of external providers, and sign posting to community initiatives.
2	A prevention‐focused approach should be adopted.	Physical activity initiatives are embedded across all CYP mental health services, including lower level providers, community services, and primary care. Examples of this may include making activity sessions accessible for CYP, who may be struggling with mental health but are not yet accessing services.
		For example, encouraging sports and activity programs in schools, through local authority and voluntary organizations, or via community outreach services.
3	Accredited exercise professionals should be used to deliver interventions where possible.	A physical activity intervention is taking place for young people who are treated in an early intervention for psychosis service delivered by a personal trainer, physiologist, or physiotherapist. This includes the fitness professional being allocated a specific role within the team where they are able to provide activity plans and guided exercise sessions directly to service users.
4	Interventions should be flexible, individualized and address the needs/motivations of the young person.	A CAMHS community‐based service is delivering an 8‐week program for young people who use their services which aims to increase activity levels to 30 min per day. The individuals are offered a range of different activities to choose from, including gym‐based exercises, running, swimming, cycling, football, martial arts, and group circuits, and assist in linking CYP with relevant opportunities for these in their local communities.
5	The setting of the intervention should fit the needs of the person who it is designed for.	A CAMHS inpatient unit is trying to encourage their service users to be more active and take part in the program. Not all the CYP are able to leave the unit and so activities need to be conducted on the ward. An external provider is introduced to the young people on the ward in advance to get to know them and then conducts the sessions three times a week onsite.

## CONCLUSION

This overview has considered why physical activity interventions are important for CYP with mental health conditions. In summary, there is an increasingly strong evidence base arising to show the benefits of using physical activity interventions to improve physical and mental health outcomes in CYP with mental disorders. However, more work needs to be done to identify the most effective ways to embed exercise interventions in mental health services for CYP and explore the long‐term impact and cost‐effectiveness of such initiatives in clinical practice. In the meantime, we present our top five recommendations for implementing exercise within CYP mental health care based on the current evidence. Now, it is critical that we establish effective ways to intervene and promote exercise at an early stage, to optimize service delivery for young people and prevent, manage, and treat the onset of physical health conditions. This will reduce the long‐term burden of comorbidities on an individual level, but also on primary, secondary, and tertiary health and social care systems. Continuing to improve the evidence base for exercise interventions in CYP, refine its implementation into mental health care, and then developing strategies for large‐scale scaling and dissemination of such interventions is all imperative, to ensure all young people are provided with the best chance to live an active and healthy lifestyle, regardless of their mental health condition, and to prevent the onset of poor physical health in these vulnerable populations.

## CONFLICT OF INTEREST

The authors declare no conflict of interest.

## ETHICS STATEMENT

No ethics approval was sought for this work as it consisted of a review of the existing literature.

## AUTHOR CONTRIBUTIONS

Rebekah Carney and Joseph Firth both contributed to the manuscript. Both authors reviewed the existing literature, extracted data, contributed to the text of the manuscript, and agreed on interpretation of the findings. Rebekah Carney provided the first written draft and Joseph Firth reviewed and edited. Both authors contributed to and approved the final submission.

## Data Availability

Data sharing not applicable to this article as no datasets were generated or analyzed during the current study.
